# The effect of preoperative sleep quality on the target plasma concentration of propofol and postoperative sleep in patients undergoing painless gastroscopy

**DOI:** 10.1186/s12871-022-01957-2

**Published:** 2023-01-07

**Authors:** Yuxue Qiu, Haitao Hou, Junxia Zhang, Xiaomei Wang, Lu Wang, Yanan Wu, Liqin Deng

**Affiliations:** 1 Department of Anesthesiology, General Hospital of Ningxia Medical University, Yinchuan, Ningxia 750004 People’s Republic of China; 2 Clinical College, Ningxia Medical University, Yinchuan, Ningxia 750004 People’s Republic of China

**Keywords:** Sleep quality, Painless gastroscopy, Propofol, Target plasma concentration, Postoperative sleep

## Abstract

**Background:**

This study aims to investigate the effect of preoperative sleep quality on the target plasma concentration of propofol and postoperative sleep in patients undergoing painless gastroscopy.

**Methods:**

Ninety-three outpatients aged 45 to 64 years with body mass index (BMI) of 18.5–30 kg/m^2^ and ASA grades of I or II, who underwent painless gastroscopy, were selected. All patients were evaluated by the Athens insomnia scale (AIS) before the painless gastroscopy. The patients were divided into two groups according to the AIS score evaluated before painless gastroscopy: normal sleep group (group N, AIS score < 4 points, 47 cases) and sleep disorder group (group D, AIS score > 6 points, 46 cases). The target-controlled infusion (TCI) of propofol (Marsh model) was used for general anesthesia, the Bispectral index (BIS) was used to monitor the depth of anesthesia, and the BIS was maintained between 50 and 65 during the painless gastroscopy. The target plasma concentration (Cp) of propofol was recorded when the patient’s eyelash reflex disappeared (T1), before the painless gastroscopy (T2), at the time of advancing the gastroscope (T3) and during the painless gastroscopy (T4), and the infusion rate per body surface area of propofol was calculated. The patient’s AIS score was followed up by telephone at day 1, day 3, 1 week, and 1 month after the painless gastroscopy to assess the postoperative sleep of the patient. The occurrence of adverse reactions during the painless gastroscopy was recorded; the patient’s satisfaction and the endoscopist’s satisfaction with the anesthesia effect were compared between the two groups.

**Results:**

Compared with group N, the Cp at each time point and the infusion rate per body surface area of propofol in group D was increased significantly (*P* < 0.05); compared with the AIS scores before the painless gastroscopy, the AIS scores of the two groups of patients were significantly increased day 1 after the painless gastroscopy (*P* < 0.05); there were no significant differences in the AIS scores of the two groups at day 3, 1 week, and 1 month after the painless gastroscopy (*P* > 0.05). There were no statistically significant differences in the occurrence of adverse reactions and the patient’s satisfaction and the endoscopist’s satisfaction with the anesthesia effect between the two groups (*P* > 0.05).

**Conclusion:**

The preoperative sleep disturbance will increase the Cp and the infusion rate per body surface area of propofol in patients undergoing painless gastroscopy. Propofol only affects the patients’ sleep for day 1 after the painless gastroscopy.

**Trial registration:**

Chinese Clinical Trial Registry (ChiCTR2100045332) on 12/04/2021.

## Background

The latest data show that the prevalence of sleep disturbance is 9.6-19.4% [1]. A report from China indicates that the proportion of adults who have experienced sleep disturbance in the past month is as high as 45.4% [2]. Sleep disturbance before surgery can lead to perioperative mental stress and obvious fluctuations in hemodynamics, which affects the safety of anesthesia [3]. Propofol is the most commonly used general anesthetic, and it is the first choice for painless gastroscopy due to its characteristics including rapid induction and complete recovery [4]. Studies have shown that continuous sedation with propofol can improve sleep in critically ill patients with mechanical ventilation in Intensive Care Unit (ICU) [5], but in healthy volunteers, propofol anaesthesia for one hour prolonged their sleep latency [6]. So far, it is not clear whether the sleep disturbance of patients undergoing painless gastroscopy affects the target plasma concentration of propofol and postoperative sleep function. Therefore, this study intends to explore the effect of preoperative sleep quality on the target plasma concentration (Cp) of propofol and the postoperative sleep in patients undergoing painless gastroscopy, provide a clinical guide for the setting of the target plasma concentration of propofol during painless gastroscopy and the recovery of postoperative sleep in patients with sleep disturbance.

## Methods

### Ethics approval

Ethical approval was obtained from the Human Research Ethics Committee of the General Hospital of Ningxia Medical University (approval NO.: 2019 − 477, Head: Prof. Dr. Hechun Xia). The study was registered in the Chinese Clinical Trial Registry (ChiCTR2100045332, Principal investigator: Yuxue Qiu, Date of registration: 12/04/2021). Written informed consent was obtained from all subjects participating in the trial, and all methods were performed in accordance with the relevant guidelines and regulations. This manuscript adheres to the applicable CONSORT guidelines.

### Study design

This study was a double-blind controlled trial performed at anaesthesia department of the General Hospital of Ningxia Medical University from April 19, 2021 to May 19, 2021. 93 outpatients aged 45 to 64 years with body mass index (BMI) of 18.5–30 kg/m^2^ and ASA grades of I or II, who underwent painless gastroscopy, were selected. The sleep states of patients who met the inclusion criteria were evaluated by the Athens insomnia scale (AIS) before the painless gastroscopy. The exclusion criteria were patients who once underwent surgery for gastrointestinal tumors, who have preoperative AIS scores of ≥ 4 points and ≤ 6 points, mental illness, poor hypertension control, cardiopulmonary dysfunction, morbid obesity, sleep apnea syndrome, gastrointestinal obstruction, severe liver or kidney dysfunction, acute upper respiratory infection, and those who need to take care of children/the elderly at night or those who work at night. Patients with the following states were eliminated during the study: endoscopic treatment is required during gastroscopy, the result of gastroscopy indicates gastric malignancy. According to the AIS scores before the painless gastroscopy, patients were divided into 2 groups (*n* = 48): normal sleep group (group N: AIS < 4 points) and sleep disorder group (group D: AIS) > 6 points). The patients were unaware of the grouping status.

#### Anesthesia

The patient fasted for 8 h and drinking was not allowed for 2 h before anesthesia. The patient was placed in the left lateral decubitus position on the examination bed, with his/her head tilted back, and dental pads were placed. The blood pressure (left upper limb), heart rate, SpO_2,_ and BIS were monitored. Oxygen was delivered at a flow rate of 6 L/min with a mask. The right upper limb venous access was established, compound sodium chloride (8–10 ml·kg^− 1^·h^− 1^) was infused intravenously. Target control infusion (TCI) of propofol was carried out using the SLGO injection pump (CP-660TCI type, Beijing SLGO Medical Technology Co., Ltd.) for general anesthesia. The initial target plasma concentration of propofol was set to 3 µg/mL, the target plasma concentration (Cp) of propofol was increased by 0.5 µg/mL every 20 s, in order to reduce BIS to 50–65 and stabilized for 1 min before advancing the endoscope. The goal of BIS is to maintain 50–65 during painless gastroscopy. If the BIS was > 65 or the patient had obvious body movement which affected the operation of the endoscopist when advancing the endoscope, the Cp of propofol was increased by 0.5 µg/mL every 20s. If the BIS was < 50 during the painless gastroscopy, the Cp of propofol was reduced by 0.5 µg/mL every 20s. The propofol was stopped infusing when removing the endoscope. If the heart rate was less than 50 bpm, atropine (0.5 mg) was administrated; if the blood pressure was lower than 20% of the baseline or the systolic pressure was less than 90 mmHg during the painless gastroscopy, phenylephrine (40 µg) or ephedrine (6 mg) was administrated; if SpO_2_ was < 90%, the inhaled oxygen concentration was increased, and mechanical ventilation was provided if necessary. The anesthetist was unaware of the patient’s preoperative sleep quality.

#### Evaluation indicators of sleep disorder

The Assens Insomnia Scale (AIS) was used for evaluating indicators of sleep disorders. The AIS is a standardized self-report questionnaire that evaluates sleep-related problems [7]. AIS consists of 8 items: sleep induction, awakenings during the night, final awakening, total sleep duration, sleep quality, well-being, functioning capacity, and sleepiness during the day. Scores range from 0 (meaning that the item in question has not been a problem) to 3 (indicating more acute sleep difficulties). A total score of < 4 points indicates no sleep disturbance, 4–6 points indicate suspected insomnia, and > 6 points indicate the presence of sleep disturbance. Preoperative sleep quality was assessed by AIS before painless gastroscopy, and AIS scores were followed up by telephone at 1 day, 3 day, 1 week, and 1 month after the painless gastroscopy. The postoperative follow-up physician was unaware of the preoperative AIS score of patients.

Observation indexes (Main observation indexes): The target plasma concentration (Cp) of propofol was recorded when the patient’s eyelash reflex disappeared (T1), before the painless gastroscopy (T2), at the time of advancing the gastroscope (T3) and during the painless gastroscopy (T4). Then, the infusion rate per body surface area of propofol was calculated. The patient’s AIS score was followed up by telephone at day 1, day 3, 1 week, and 1 month after the painless gastroscopy to assess the postoperative sleep of the patients. Secondary observation indexes: the procedure time of gastroscopy, the patient’s recovery time (from the time of withdrawal of drugs to the time that the patient was able to clearly say his/her name, age, and birthday) and the orientation recovery time (time between the withdrawal of drugs and the orientation of the patient to time, place, and person) were recorded; the occurrence of adverse reactions such as coughing, intraoperative body movement, hypotension (the blood pressure decreased by over 20% of the basic value or the systolic pressure was < 90 mmHg), bradycardia (heart rate < 60 bpm), respiratory depression (apnea for more than 15 s, or SpO_2_ < 90%), and intraoperative awareness was recorded. The patient’s satisfaction and the endoscopist’s satisfaction was evaluated: the visual analog scale (VAS) was used to evaluate the satisfaction of the patient and that of the endoscopist (unsatisfied: 0–3; basically satisfied: 4–6; satisfied: 7–10).

#### Statistical analysis

According to the results of our pre-experiment, when the eyelash reflex disappeared, the propofol Cp of the normal sleep group was 3.75 ± 0.42 µg/mL, and that of the sleep disturbance group was 4.10 ± 0.52 µg/mL (α = 0.05, 1-β = 0.9, two-sided test). PASS 11 software was used to calculate the sample size, N1 = N2, which suggested that at least 39 cases should be included in each group. The expected follow-up dropout rate was 20%, so 48 cases were finally included in each group. SPSS 23.0 software was used for statistical analysis. Normally distributed measurement data were expressed as mean ± standard deviation ($\overline{\varvec{x}}$±s), and the *t*-test was used for group comparison. The repeated measures analysis of variance was used for intragroup comparisons of AIS scores at different time points. The Wilcoxon rank-sum test was used for the analysis of skewed measurement data. Count data were expressed as the number of cases or percentage (%), and the χ^2^ test or Fisher’s exact test was used for the comparison between groups to calculate the exact probability. *P* < 0.05 was considered statistically significant.

## Results

The study initially enrolled 172 patients and excluded 76 patients. These 76 patients included 51 patients with AIS scores of ≥ 4 points or ≤ 6 points, 1 patient who underwent gastrointestinal tumor surgery, 3 patients with poorly controlled hypertension, 1 patient with cardiopulmonary insufficiency, and 2 patients with sleep apnea syndrome, 2 patients with acute upper respiratory tract infection, and 16 patients who need to take care of children/elderly people or night shifts at night. 1 patient in the group N (the patient underwent gastroscopic polypectomy) and 2 patients in the group D (1 patient underwent gastroscopic polypectomy and 1 patient was diagnosed with gastric cancer by gastroscopy) were eliminated during the study. Finally, 93 patients were included, 47 patients in group N, and 46 patients in group D. There were no statistically significant differences between two groups of patients in the gender, age, BMI, years of education, painless gastroscopy time, recovery time, and orientation recovery time. (*P* > 0.05, Table 1).

**Table 1:** Comparison of general information of the two groups of patients (‾*x ± s*)

Groups	Cases	M/F (cases)	Age (years)	BMI (kg/m^2^)	Years of education (≤ 6 / >6 to ≤ 9 / >9 to ≤ 12 / >12) (years)	painless gastroscopy time (min)	Recovery time (min)	Orientation recovery time (min)
Group N	47	18/29	53.5 ± 6.0	23.9 ± 2.5	12/13/12/10	4.5 ± 1.6	9.1 ± 2.1	10.9 ± 2.3
Group D	46	17/29	53.9 ± 5.0	23.4 ± 3.2	14/12/10/10	4.3 ± 1.2	8.8 ± 2.8	10.8 ± 2.9

**Table 2:** Comparison of target plasma concentration and the infusion rate per body surface area of propofol between two groups of patients (‾*x ± s*)

Groups	Cases	T_1_ (µg/mL)	T_2_ (µg/mL)	T_3_ (µg/mL)	T_4_ (µg/mL)	The infusion rate per body surface area [mg/(min·m^2^)]
Group N	47	3.8 ± 0.4	4.6 ± 0.4	4.7 ± 0.4	4.6 ± 0.4	14.7 ± 2.4
Group D	46	4.0 ± 0.5	4.8 ± 0.4	4.9 ± 0.5	4.8 ± 0.5	16.2 ± 2.2
*T* value		-2.329	-2.464	-2.446	-2.511	-3.190
*P* value		0.022	0.016	0.016	0.014	0.002

Compared with the group N, the Cp of propofol at four different time points, and the infusion rate per body surface area of propofol increased significantly in the group D (*P* < 0.05, Table 2)

**Table 3:** Comparison of AIS scores between the two groups of patients at different time points (‾*x ± s*)

Groups	Cases	Preoperative AIS	AIS of day 1 after painless gastroscopy	AIS score of day 3 after painless gastroscopy	AIS score of 1 week after painless gastroscopy	AIS score of 1 month after painless gastroscopy
Group N	47	1.9 ± 0.9	3.0 ± 1.1^a^	2.1 ± 0.9	2.1 ± 1.0	2.0 ± 1.0
Group D	46	10.7 ± 3.2	12.0 ± 3.5^a,b^	11.1 ± 3.5^b^	11.0 ± 3.2^b^	11.0 ± 3.4^b^

*Compared with preoperative AIS, ^a^*P* < 0.05, and compared with Group N, ^b^*P* < 0.05. Compared with the AIS scores before the painless gastroscopy, the AIS scores of the two groups of patients were significantly increased at day 1 after the painless gastroscopy (*P* < 0.05, Table 3); compared with the N group, the AIS scores of the group D were significantly higher at day 1, day 3, 1 week and 1 month after the painless gastroscopy (*P* < 0.05, Table 3)

The incidences of coughing during the painless gastroscopy in the group N and the group D were 14.9% and 17.4% respectively; the incidence of intraoperative body movement during the painless gastroscopy in group N and D were 21.3% and 23.9% respectively; there were no significant differences between the two groups of patients in the incidences of coughing and intraoperative body movement (*P* > 0.05). There was no occurrence of hypotension, bradycardia, respiratory depression, or intraoperative awareness in the two groups of patients. There were no statistically significant differences between two groups in the patient’s satisfaction and the endoscopist’s satisfaction (*P* > 0.05, Table 4).

**Table 4:** Comparison of the patient’s satisfaction and the endoscopist’s satisfaction between the two groups of patients [case (%)]

Groups	Satisfaction score (points)	Patient’s satisfaction	Endoscopist’s satisfaction
Group N (7 cases)	10	27 (57.4%)	31 (66.0%)
	9	14 (29.8%)	14 (29.8%)
	8	4 (8.5%)	2 (4.3%)
	7	2 (4.3%)	0 (0.0%)
Group D (46 cases)	10	34 (73.9%)	34 (73.9%)
	9	9 (19.6%)	10 (21.7%)
	8	2 (4.3%)	2 (4.3%)
	7	1 (2.2%)	0 (0.0%)
*P* value		0.481	0.799

## Discussion

This study used the internationally recognized self-assessment psychometric instrument designed for quantifying sleep difficulty [8], the Athens Insomnia Scale (AIS), to assess the sleep quality of patients. It has been reported that the reliability of AIS is 0.89 and the validity is 0.9 [7]. The AIS consists of 8 items, the score of each item ranges from 0 (meaning that the item in question has not been a problem) to 3 (indicating more acute sleep difficulties). Higher AIS scores indicate greater severity of insomnia symptoms [9]. A total score of < 4 points indicates no sleep disturbance, 4–6 points indicate suspected insomnia, and > 6 points indicate the presence of sleep disturbance [10]. The results of this study showed that the Cp and the infusion rate per body surface area of propofol in patients undergoing painless gastroscopy, who had sleep disturbances before the painless gastroscopy were increased significantly; the sleep function of patients undergoing painless gastroscopy using propofol, regardless of whether they had sleep disturbances before the painless gastroscopy, was decreased to the same extent day 1 after the painless gastroscopy, but there was no change in sleep function recovery day 3, 1 week, and 1 month after the painless gastroscopy.

Due to the large individual differences in the pharmacokinetics and pharmacodynamics of propofol, the conventional methods of drug administration cannot well meet the needs of individual sedation in clinical practice. Therefore, individualized monitoring of the depth of anesthesia and sedation to control of the dosage has become more and more important. Doi, et al. [11] observed that BIS values between 64 and 80 are the transition from loss to return of consciousness. Kears, et al. [12] reported that the BIS value for predicting 95% of people in an unconscious state was 52, and the results of our preliminary experiment showed that the BIS value of the appropriate depth of anesthesia for patients undergoing painless gastroscopy was between 50 and 65. Therefore, in this study, we set the BIS value between 50 and 65 to maintain the appropriate depth of sedation without generating intraoperative awareness, as well as to retain certain stress responses. Our results also confirmed that this anesthesia method had few and mild adverse reactions during the painless gastroscopy of the two groups of patients, the patient’s satisfaction and the endoscopist’s satisfaction was high, and the anesthesia effects of the two groups of patients were fine.

There are many factors that affect the Cp of propofol in patients undergoing painless gastroscopy, including age, gender, weight, the intensity of noxious stimulation, drug interactions, etc. [13–17]. In addition, there are also many factors that affect postoperative sleep function, such as patient age, surgical factors, anesthesia factors, postoperative pain, environmental and psychological factors, etc. [18–21]. In order to maximize the control of various factors affecting the propofol Cp and postoperative sleep function, we only focused on the impact of the preoperative sleep quality, selected only outpatients undergoing gastroscopy with propofol anesthesia, the general data of the two groups of patients were comparable.

γ-aminobutyric acid (GABA) is the main inhibitory neurotransmitter in the central nervous system, which is widely distributed in the human central nervous system. GABA receptors are subdivided into type A and type B, which have inhibitory effects in the central nervous system. Propofol mainly acts on type A receptors, enhancing the reaction between GABA and type A receptors, resulting in decreased excitability of neural networks and playing the role of anesthesia. In addition, N-methyl-D-aspartic acid (NMDA) is an important central excitatory glutamate in the body, and propofol can inhibit the transmission of excitement by weakening the binding of NMDA and its receptors [22]. The level of GABA decreased and glutamate increased in patients with sleep disorders [23]. Therefore, compared to the normal sleepers, higher concentrations of propofol are needed to achieve the same anesthetic effect. The results of our study showed that the Cp and the infusion rate per body surface area of propofol in painless gastroscopy patients with sleep disorders were significantly increased. This results suggests that clinical anesthesiologists need individual administration of propofol during general anesthesia for patients with different sleep quality in order to achieve satisfactory anesthesia effect.

Many literatures have reported that patients undergoing surgery are prone to sleep disorders 1 night after surgery due to the combined action of many factors (such as lights and noise in the ward, surgical trauma, postoperative pain, anaesthetics, interference of night nurse care, etc.). In order to avoid the interference of these factors on postoperative sleep, we only selected outpatient patients who underwent painless gastroscopy. Different from surgery, patients underwent painless gastroscopy have less trauma and psychological pressure, no obvious discomfort after gastroscopy. So the results can better reflect the relationship between propofol and sleep disruption. Kushikata et al. have confirmed that propofol can affect the secretion of orexin and melatonin [24], and interfere with the body’s postoperative sleep-wake cycle [25]. Propofol is an ultra-short-acting general anesthetic with rapid metabolism, which induces complete awakening at the end of anesthesia. But the fading of the anesthetic effect of propofol does not mean it has been completely cleared from the body. Schuttler et al. found that the pharmacokinetic characteristics of propofol were in line with the three-compartment model, and its elimination half-life (t_1/2β_) was 335 min. After 4 to 5 half-lives of propofol in vivo, it could eliminate 93.75–96.88% [26]. It has been reported that propofol based intravenous general anesthesia can affect the sleep quality of patients undergoing laparoscopic surgery 3 days after surgery [27]. Another study showed that propofol and sufentanil anesthesia can affect the sleep quality of patients 7 days after diagnostic upper gastrointestinal endoscopy [28]. But many sedative and analgesic drugs were used in these sdudies, it could not be clear which drugs caused the results. So our study took a longer period as the observation endpoint in order to observe whether the use of propofol anesthesia alone would cause long-term effects on patients’ sleep. And we also wanted to compare the difference in sleep quality over a longer period after painless gastroscopy between the two groups. This study shows that excluding surgical factors, environmental and psychological factors, propofol affects the patient’s sleep only day 1 after painless gastroscopy regardless of their preoperative sleep quality. And the sleep quality of patients in both groups returned to the preoperative level at day 3, 1 week and 1 month after painless gastroscopy. The results were also consistent with the pharmacokinetics of propofol. In other words, the disruption of sleep by propofol is temporary.

The limitations of this study are as follows. First, although AIS is a reliable scale for evaluating sleep, its subjectivity is obvious. If objective evaluation indicators (such as polysomnography or motion recorder) are available too, it would be better. But for outpatients, these indicators are less likely to be used. Second, we only studied middle-aged people of normal weight, the results may not be applicable to other age groups and obese patients. Third, although we avoided many factors that affected patients’ sleep in the study design and the baseline characteristics of the two groups were consistent, but we did not take into consideration of the multiple confounders (such as anxiety and depression) when the analysis was done. Forth, this study did not include patients undergoing painless gastroscopy with suspected insomnia. The effects of propofol on the Cp and postoperative sleep in this population during painless gastroscopy need to be further studied.

In conclusion, the Cp and the infusion rate per body surface area of propofol increase in patients with preoperative sleep disturbance during painless gastroscopy; propofol only affects the patients’ sleep quality for day 1 after the painless gastroscopy.

## Data Availability

All data generated or analyzed during this study are included in this published article.

## Abbreviations

BMI: Body mass index
BIS: Bispectral index
TCI: Target-controlled infusion
VAS: Visual analog scale
GABA: γ-aminobutine acid
CNS: Central nervous system
